# Comparison of Two Derivatization Methods for the Analysis of Fatty Acids and *Trans* Fatty Acids in Bakery Products Using Gas Chromatography

**DOI:** 10.1155/2014/906407

**Published:** 2014-02-25

**Authors:** Jumat Salimon, Talal A. Omar, Nadia Salih

**Affiliations:** School of Chemical Sciences & Food Technology, Faculty of Science and Technology, Universiti Kebangsaan Malaysia, 43600 Bangi, Selangor, Malaysia

## Abstract

Two different procedures for the methylation of fatty acids (FAs) and *trans* fatty acids (TFAs) in food fats were compared using gas chromatography (GC-FID). The base-catalyzed followed by an acid-catalyzed method (KOCH_3_/HCl) and the base-catalyzed followed by (trimethylsilyl)diazomethane (TMS–DM) method were used to prepare FA methyl esters (FAMEs) from lipids extracted from food products. In general, both methods were suitable for the determination of *cis*/*trans* FAs. The correlation coefficients (*r*) between the methods were relatively small (ranging from 0.86 to 0.99) and had a high level of agreement for the most abundant FAs. The significant differences (*P* = 0.05) can be observed for unsaturated FAs (UFAs), specifically for TFAs. The results from the KOCH_3_/HCl method showed the lowest recovery values (%*R*) and higher variation (from 84% to 112%), especially for UFAs. The TMS-DM method had higher *R* values, less variation (from 90% to 106%), and more balance between variation and %RSD values in intraday and interday measurements (less than 4% and 6%, resp.) than the KOCH_3_/HCl method, except for C12:0, C14:0, and C18:0. Nevertheless, the KOCH_3_/HCl method required shorter time and was less expensive than the TMS-DM method which is more convenient for an accurate and thorough analysis of rich *cis*/*trans* UFA samples.

## 1. Introduction

Oils and fats in foods are composed of four different types of FAs: polyunsaturated, monounsaturated, saturated, and TFAs [1]. Naturally, all unsaturated FAs in vegetable oils are in the *cis* form, whereas a large proportion of unsaturated FAs isomerize to their TFA counterparts during the industrial hydrogenation of vegetable oils [2]. Therefore, dietary fats made with fully and/or partially hydrogenated oils, which are used in foods to improve texture and stability for a longer shelf life, contain TFAs [3, 4]. Likewise, bakery products made with hydrogenated oils and fats, such as biscuits, cakes, cookies, crackers, and breads, contain TFAs [5, 6].

Results published in recent years indicate the importance of food FA composition in human nutrition and health [5, 7]. In general, it is recommended to increase the intake of n-3 polyunsaturated FAs (PUFAs) and to decrease the intake of saturated fatty acids (SFAs) and TFAs because TFAs affect cholesterol levels in much the same ways as saturated and *trans* fats increase your risk of developing coronary artery and heart diseases [8, 9]. This association between the dietary consumption of some FAs and increased risk of some diseases has led to the implementation of new regulations that require the declaration of FAs, including TFA content, on the labels of conventional foods and dietary supplements in several countries [2, 3, 10]. Therefore, it is important to have accurate and precise techniques for the identification and quantification of FAs and TFAs in foods of natural origin or in foods formed during the processing of fats and oils [1, 11] that is performed due to consumer demand for improved fat quality in foods [12].

In recent years, GC has been used for the separation and analysis of geometric and positional isomers. Although GC/mass spectrometry and other technical methods have been developed to quantitate C8–C26 chain-length FAs, the GC analysis of FAs with FID remains the most frequently used method [1, 13–17]. The quantification of FAs in fats and oils by GC involves transforming the analytes into more volatile and nonpolar derivatives after extracting the lipids from the food product before GC analysis [14]. The most important stage for the GC-FID determination of FAs is sample preparation, which usually requires derivatization of the FAs to increase the volatility of the substances to improve separation and to reduce tailing [18]. Moreover, the speed of analysis, sensitivity, and accuracy are important parameters in GC that may be improved with derivatization [18, 19].

Sample preparation, including the derivatization of FAs, has been carefully reviewed by several authors [19–21]. The most commonly used method for the determination of FAs is conversion of the FAs into their corresponding methyl esters (FAMEs). Many different methylation approaches have been described in the literature, and some methods have been established for preparing FAMEs from lipids extracted from various food samples: acid- or base-catalyzed transmethylation, borontrifluoride (BF_3_) methylation after hydrolysis, methylation with diazomethane, and silylation [18–20, 22–24]. In general, these methods involve two steps: first, the samples are heated with sodium hydroxide in methanol and, second, the free FAs (FFAs) are esterified with methanolic BF_3_ [23] or methanolic KOH [24]. However, each method has its own advantages and disadvantages [16, 25].

In general, the base-catalyzed method for the direct transesterification of lipids has been reported to be more applicable for nutrition analysis because it is easy to use and uses less aggressive reagents than other methods [22, 24, 26]. However, this method has resulted in poor recoveries of FAMEs because FFAs might remain partially unreacted [27] and because FFAs are not methylated under these conditions [26]. Therefore, some studies have suggested that the repeatability, recovery with low variation, and the highest concentration detected are improved for the most abundant FAs when the combined base- and acid-catalyzed method is used compared to the base- or acid-catalyzed methods alone [20, 26, 28, 29]. Nevertheless, using acid-catalyzed methods is usually undesirable because it is likely to lead to changes in the configuration of the double bond characteristics and to produce artifacts [20, 25, 30].

An alternative method used by a number of laboratories to improve the accuracy of analysis is base hydrolysis followed by methylation of the resulting FFAs with diazomethane; however, the disadvantage of this method is that diazomethane needs precautions during extraction [21, 31, 32]. In contrast, the esterification by TMS-DM has been reported to be a convenient alternative to diazomethane because it is safer to handle and does not produce artifacts [33, 34]. Furthermore, methylation by TMS-DM after the saponification process has been shown to be more accurate for *cis*/*trans* PUFA analysis in seafood [31] and conjugated linoleic acid (CLA) isomers in ruminant meat tissues [32] when compared to other methylation reagents. However, the hydrolysis or presence of trace water leads to poor recoveries of FAMEs [16, 27].

There is a need to investigate the concentration of FA and TFA isomers in all lipid fractions from food fats and their products, such as biscuits, cakes, crackers, wafers, and bread, to monitor the low levels of FAs and TFAs and to control labeling authenticity. Therefore, it is possible to apply the advantages of sodium methoxide (NaOCH_3_) as a useful reagent for the fast transformation of FAs into FAMEs [18, 35] along with using the TMS-DM reagent for the complete methylation of all FFAs, which can be more reliable and produce a higher accuracy. In the current study, to verify the accuracy of measuring the concentrations of FAs and TFAs in food fats of bakery products, the repeatability and recovery using a method based on the derivatization of lipid extract by base-catalyzed followed by TMS-DM were compared with the combined base- and acid-catalyzed methylation method (KOCH_3_/HCl). In addition, the advantages, disadvantages, and applicability to determine the complex mixture of FAs and TFAs in various types of bakery products are discussed.

## 2. Materials and Methods

### 2.1. Standards and Reagents

Nine FA and FAME standards (C12:0, C14:0, C16:0, C18:0, C18:1, C18:1t9, C18:2, C18:2t9,12, and C18:3) were purchased from Fluka (purity; ≥99% (GC); Sigma-Aldrich, Germany), the internal standard (IS) C15:0 (Pentadecanoic acid) was purchased from Sigma (Sigma-Aldrich, Germany), and the purity of all reagents was greater than 99%. All chemicals (methanol, toluene, glacial acetic acid, hydrochloric acid potassium hydroxide, and sodium hydroxide) were of analytical reagent grade and purchased from Systerm (Systerm, Malaysia) except for n-hexane, which was of higher purity (Systerm, Malaysia, for GC, ≥99%). The esterifying agent TMS-DM (2 M) in n-hexane was purchased from Sigma (Sigma-Aldrich, Germany).

### 2.2. Food Samples

Eight commercial food items were used for analysis and comparison in this study. The samples included different bakery and fast-food products, such as crackers, bread with filling, cakes, wafers, cookies, and biscuits, as these products mainly contain FAs and TFAs. The samples were purchased from several Malaysian local supermarkets, including national and imported brands, and all of those samples were coded with a letter (from A to H).

### 2.3. Sample Preparation and Lipid Extraction

Each sample was ground and placed in an oven at 50°C until complete dryness before analysis. The total lipids were extracted using the Soxhlet Method for cereal fats [28]. Approximately 10 g of homogenized sample was weighed into a cellulose extraction cartridge, and the Soxhlet apparatus containing the cartridge was fitted to a distillation flask containing 150 mL of n-hexane with (50 ppm) butylated hydroxytoluene (BHT) and a few antibumping granules. After 3 hours, the mixture was dried with Na_2_SO_4_ and filtered through fluted filter paper. The oil was recovered after stripping the solvent in a rotary evaporator. Finally, the extracted lipids were dried under nitrogen (N_2_), weighed,and stored at −20°C until analysis.

### 2.4. Preparation of Fatty Acid Methyl Esters (FAMEs)

**Figure 1:**
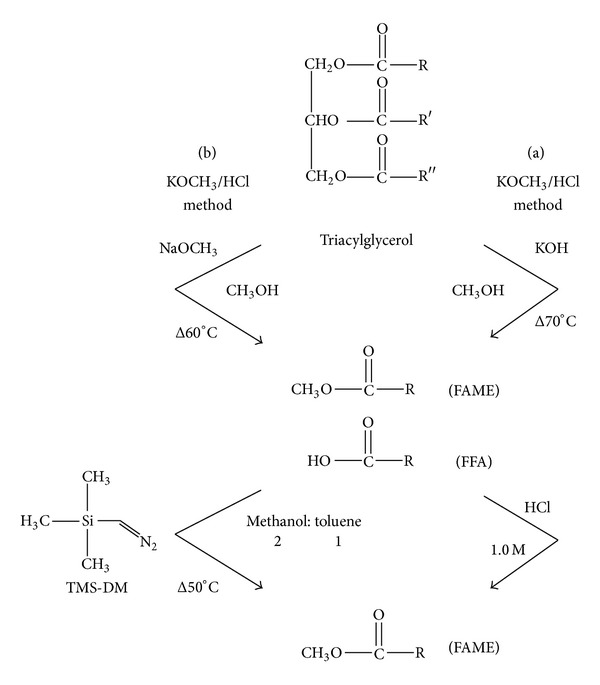
Diagram for the procedures of the method (a) (KOCH_3_/HCl) and method (b) (TMS-DM).

After Soxhlet extraction, all lipid extracts were methylated and converted into FAMEs using two different methylation methods. Approximately 0.15 g of each fat extract (in triplicate) was transferred to a screw-cap test tube (10 mL), and 1 mL of a solution containing 10 mg/5 mL (C15:0) in methanol was added as an IS. The mixtures were reduced to dryness under nitrogen (N_2_) before derivatization using two different methodologies (Figure 1), and the procedures were performed as described in the following sections.

#### 2.4.1. Base-Catalyzed Followed by the Acid-Catalyzed Method (KOCH_3_/HCl) 

The mixtures were redissolved in 2 mL of n-hexane, and 1 mL of 2 M methanolic KOH solution was added to the samples. The tubes were capped and vigorously shaken for 30 s and boiled for 2 min in a water bath at 70°C. Then, 1.2 mL of HCl (1.0 M) was added and the solution was gently stirred. After phase separation, 1 mL of n-hexane was added. The upper phase containing the FAMEs was transferred into an analysis vial, and 1.0 *μ*L of the solution was injected into the GC-FID.

#### 2.4.2. Base-Catalyzed Method Followed by TMS-DM 

The mixtures were redissolved in 2 mL of n-hexane, and 1 mL of 2 M NaOCH_3_ was added. The content was placed in a water bath at 60°C for 5 min. Drops of concentrated glacial acetic acid were added to each tube to neutralize the NaOH. The samples were reduced to dryness under N_2_ and dissolved in 1 mL of methanol : toluene (2 : 1 vol.). Next, TMS-DM was added in a molar excess of 2 M in n-hexane (100 *μ*L) at 50°C for 10 min without capping the tubes. Drops of glacial acetic acid were added until the yellow color disappeared to remove any unreacted TMS-DM, and the reaction mixture was diluted with 1 mL of a 0.5% NaCl solution. To extract the FAMEs, 1 mL of n-hexane (containing 50 ppm BHT) was added and the tubes were vortexed for 30 s. After the solution settled, the organic layers containing the methyl esters were transferred to a vial for GC.

### 2.5. GC Analysis

The samples were manually injected (1 *μ*L) into a gas chromatograph (Shimadzu, GC-17A, Kyoto, Japan) equipped with a flame ionization detector (FID) for separation and quantification of the FAMEs. The analysis was performed using a BPX-70 fused silica capillary column (30 M, 0.25 mm i.d., 0.2-*μ*m film thickness; Melbourne, Australia). The run was under an optimized temperature program as follows: initial column temperature was 100°C and was programmed to increase at a rate of 10°C min^−1^ up to 160°C and then at 3°C min^−1^ up to 220°C. This temperature was maintained for 5 min, increased at 10°C min^−1^ to a final temperature of 260°C, and held for 5 min. The injector and detector temperatures were 260°C and 280°C, respectively. Helium was used as the carrier gas at a flow rate of 1 mL min^−1^ with a split ratio of 60 : 1.

### 2.6. The General Measurement Procedures

#### 2.6.1. Calibration and Quantification

A standard mixture, containing all FAMEs and the IS (C15:0), was used to prepare five working standard sets by diluting the stock solution with n-hexane. Calibration curves were constructed from the analysis of the working standards in triplicate using the same GC conditions as those used for quantitative purposes. According to the Multiple Point I.S. method [36], a calibration plot of each compound was run by using the ratio of the peak area of the FAME standards to the peak area of the IS against the ratio of the concentration of the FAME standards to the concentration of the IS.

The retention times of the selected FAME standards were used to identify individual FAs and TFAs in food fat samples. The concentration of FAMEs in the samples was determined using the area ratio and calibration plots. For both methods, the absolute and relative contents of 9 FAs were calculated from all 8 bakery products.

#### 2.6.2. Comparison of the Precision and Accuracy

The study included a comparison between both methods in terms of precision and accuracy for each analytical procedure according to guidelines for the validation of chromatographic methods [37]. The precision of the methods was checked through the repeatability (intraday) and reproducibility (interday), and both values were expressed as relative standard deviation (RSD%). The values for intraday RSD were calculated using the measured data from a single day, and interday RSD values were calculated using the measured data from three successive days.

The accuracy of both methods was verified using a recovery assay. The recovery was established by spiking the extracted fats of four selected samples with a FA standard at two different concentrations (Std1 and Std2) and assaying the sample in triplicate. The concentrations of the FAs in the nonspiked samples were subtracted from the concentrations in the spiked samples, and the recovery percentages (*R*%) were calculated by dividing the calculated concentrations by the expected concentrations.

### 2.7. Statistical Evaluation

A paired *t*-test was used to compare the differences between the mean values for the content of each FA measured using both methods (significance level *P* ≤ 0.05). To evaluate the precision of both methods, the intraday and interday RSD % values for each component of all samples were calculated, and the calculation of the means and standard deviations (SD) was performed using Microsoft Excel (Professional Edition 2007; Microsoft Corporation, Redmond, WA, USA). The correlation coefficients (*r*) between both methods were calculated for each FA as a measure of concordance.

## 3. Results and Discussion

### 3.1. Analysis of Selected Samples

#### 3.1.1. Identification of FAMEs

**Figure 2:**
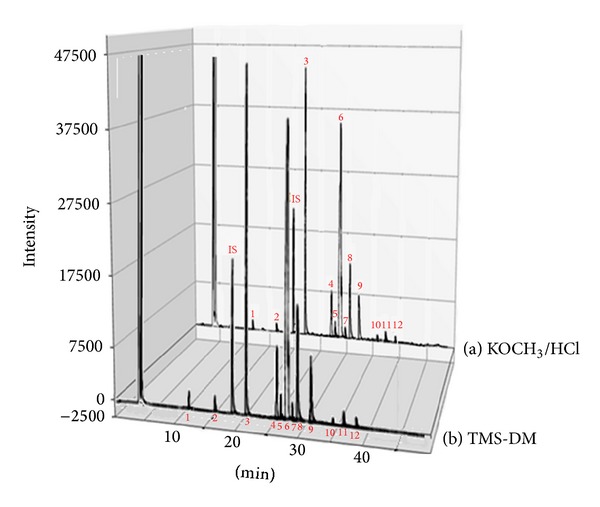
GC-FID chromatograms of the total FAs for a sample of bakery products (biscuit) derivatized by the KOCH_3_/HCl method (a) and TMS-DM method (b) 1 = C12:0; 2 = C14:0; 3 = C16:0; 4 = C18:0; 5 = C18:1 t9; 6 = C18:1; 7 = 18:2 9t,12t; 8 = C18:2; 9 = C18:3; 10 = C20:0; 11 = C22:0, 12 = C24:0, IS = C15:0.

As research on *cis*/*trans* UFAs and other FAs in food products becomes more popular, it is essential to provide correct information about the composition and the performance of quantitative analysis using the proper application of the methylation procedure [30]. Therefore, in this current study, eight different bakery and fast-food products with varying FA and TFA contents were analyzed using two derivatization procedures (described above) to prepare FAMEs for GC analysis in triplicate to compare the two methods and to discuss their advantages and disadvantages. FAMEs in the samples were identified by conducting a comparison of similar peak retention times (Rt) using pure FAME standards.  Figure 2 shows typical GC-FID chromatograms of total FAs in a sample of biscuits determined using both methylation procedures as previously outlined.

The chromatograms for both methods show that all peaks representing all components were well resolved with a good separation between the FA and TFA peaks within 37 min, and this result indicates that peak overlap was not affected by the peaks of the major constituents in both methods, which is unlike some of the chromatograms produced by other methods [38]. However, it is possible that there are some relative differences between the areas of some FA peaks for both methods.

Furthermore, no strange peaks or artifacts that interfered with the FA chromatographic peaks were detected in both chromatograms, although this result was more obvious in the chromatogram of the TMS-DM method. In general, this result also confirms earlier reports stating that TMS-DM did not produce any methoxy artifacts associated with the base catalysts [27, 32, 39, 40].

#### 3.1.2. Quantification of FAMEs

For both methods, the concentrations of all nine FAs studied, including TFAs, were analyzed and calculated for all eight food samples in absolute (g/100 g) and relative (w/w percentage) contents. Tables 1 and 2 present the means of the absolute (g FA 100 g^−1^ sample) and relative (% of total identified FA) FA contents in all samples using the base-catalyzed followed by the acid-catalyzed method (KOCH_3_/HCl) and base-catalyzed method followed by methylation with TMS-DM, respectively.

As observed in Tables 1 and 2, higher concentrations for all *cis* and *trans* FAs were observed following the TMS-DM method compared to the KOCH_3_/HCl method, whereas C12:0 and C16:0 were at slightly lower concentrations for some of the samples (no significant differences) following the TMS-DM method than for the KOCH_3_/HCl method. Less significant differences between the two methods were observed for the absolute and relative contents of *cis*-UFAs for most of the samples. The relative proportions also show that significant differences between the two methods are found for the TFAs. All other FAs showed no statistically significant differences for the relative composition between the two derivatization methods.

On the other hand, most researches which are interested in the analysis of fats in bakery products and food samples usually focus on a couple of major isomers: C18:1 *cis*-9 with C18:1 *trans*-9 as well as C18:2 *cis*-9,12 with C18:2 *trans*-9,12 [28, 41–43]. The current study found that the KOCH_3_/HCl and TMS-DM methods gave results that were significantly different for these FAs and contained greater significant differences for C18:1 *trans*-9 and C18:2 *trans*-9,12 and less significant differences for C18:1 *cis*-9 and C18:2 *cis*-9,12. However, in each case, the TMS-DM method rendered a higher percentage, which conforms to the higher concentration of detectable components.

### 3.2. The Correlation Coefficient

The correlation coefficients between both methods were calculated for each FA.  Table 3 presents the correlation coefficients between both methods for all FAs studied.

**Figure 3:**
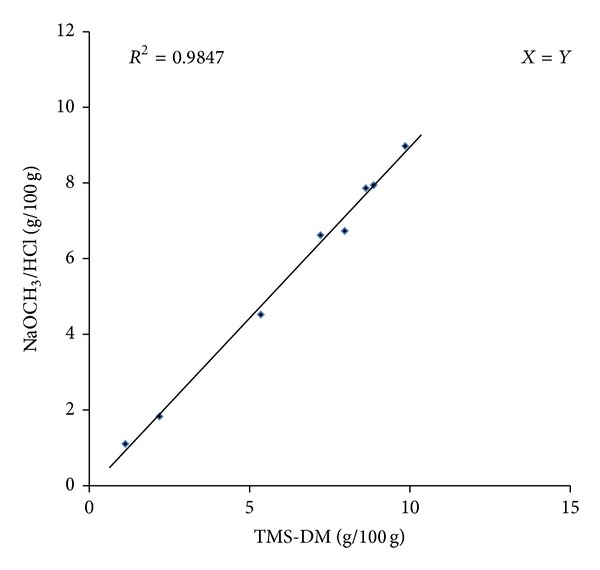
Comparison of C18:1 *cis*-9 measurements (g/100 g) by GC-FID based on the KOCH_3_/HCl and TMS-DM derivatization procedures.

**Figure 4:**
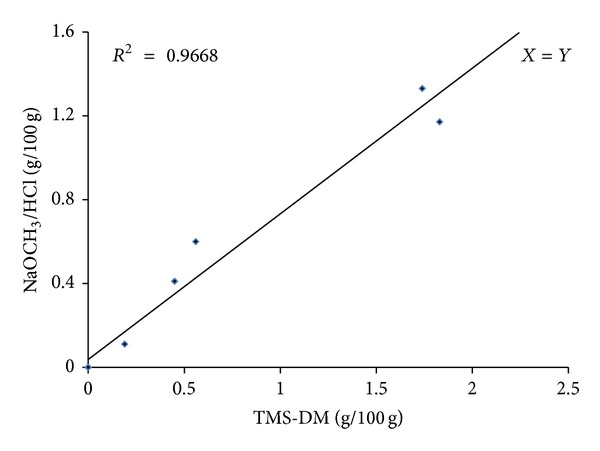
Comparison of C18:1 *trans*-9 measurements (g/100 g) by GC-FID based on the KOCH_3_/HCl and TMS-DM derivatization procedures.

For two of the FAs, C18:1 *cis*-9 and C18:1 *trans*-9, all 8 mean measurements of the TMS-DM and KOCH_3_/HCl methods were plotted (*X* = *Y*) to demonstrate the degree of agreement between the two methods, as shown in Figures 3 and 4, respectively. The correlation coefficient was 0.98 for C18:1 *cis*-9 and 0.96 for C18:1 *trans*-9.

For the low-level FAs (C14:0, C18:2 *trans*-9,12 and C18:3), the correlation coefficients (0.89, 0.86 and 0.89, resp.) between the methods were relatively small. In addition, a high level of agreement between the two methods was observed for two of the most abundant FAs (C16:0 and C18:1 *cis*-9) where the correlation coefficients were high (0.99 and 0.98, resp.).

### 3.3. Comparison of Accuracy

To evaluate the accuracy for both procedures, the recovery percentage (%*R*) values were calculated. The (%*R*) values of both methods and FAs were established from the complete analysis (in triplicate) of four food samples fortified with FA standards at two levels (std1 and std2). In Table 4, mean values of *R* for both methods are presented.

As observed in Table 4, the lowest *R* values at the two studied levels were those for the KOCH_3_/HCl method. However, for most samples, the *R* values in this method were slightly higher for C12:0, C16:0, and C18:0. The values decreased when lower concentrations were used. Moreover, these data show a high range of values obtained from this method (between 84 and 112). On the other hand, the TMS-DM method showed higher *R* values except for some saturated FAs in most of the samples, which showed *R* values slightly lower than the other method. Moreover, an increased level of homogeneity was observed because the values ranged between 90% and 106% at the two levels. Accordingly, the KOCH_3_/HCl method showed the lowest recovery values and highest variation. These results suggest that the conditions of this process might not be sufficient to methylate all lipids.

### 3.4. Comparison of Precision

The repeatability (intraday RSD) and reproducibility (interday RSD) of the replications on real samples were used to measure the precision of both methods. The repeatability of both methods was established from four (*n* = 4) complete analyses of each sample under the same conditions on one day, and the reproducibility was established from three (*n* = 3) complete analyses of each sample repeated for three consecutive days. The repeatability and reproducibility data are shown in Tables 5 and 6, respectively, and the results are expressed as a relative standard deviation (RSD, %).

For most samples except for TFAs, the KOCH_3_/HCl method showed intraday RSD values lower than 5%. The C18:1 *trans*-9 and C18:2 *trans*-9,12 had greater relative variation. The interday RSD for this method had values under 6% except for TFAs (especially for C18:2 *trans*-9,12), which showed greater values.

The TMS-DM method had the lowest RSD with intraday RSD values less than 4%. Most of the FAs and TFAs studied had the lowest RSD variation values, which ranged between 0.32% and 3.01%, except for C14:0 and C18:0. Most of the interday RSD values for the TMS-DM method ranged between 1% and 5%. The highest values for most of the samples were those observed for C14:0 and C18:3.

In general, the results from the KOCH_3_/HCl method showed the lowest recovery values, especially for *cis*/*trans* UFAs, and the highest intraday and interday variation values for TFAs. The rest of the FAs studied had acceptable variation values. These results match to some extent with those by Neo et al. [6] and Phillips et al. [23]. Although the base-catalyzed method for the direct transesterification of lipids is more applicable for routine analysis of some food samples because it is easy to use and does not isomerize *cis*/*trans* UFAs [18], FFAs and some lipid classes, such as those found in sphingolipids, are not methylated under these conditions [30]. Therefore, this method has resulted in poor recoveries of FAMEs [27]. Some studies have proven that the combined base-catalyzed method and acid-catalyzed method compared to the base-catalyzed method alone has led to better results in accuracy and precision of the analysis by improving the repeatability and *R* values [20, 26, 28]. Nevertheless, other studies that used the acid-catalyzed method have indicated that BF_3_, HCl, and other acidic catalysts will change the double-bond configuration of *cis*/*trans* FAs (e.g., octadecadienoic isomers; CLA). Thus, acidic catalysts are not recommended for lipid samples that have a mixture of these structures, such as bakery, dairy, and ruminant meat products [30]. In addition, it has been reported that, when using a paste date or concentrated reagent of acids, the production of artifacts as well as the loss of PUFAs may result [18, 20]. In summary, the use of HCl in methanol and other acidic catalysts is not recommended because the reactions take a long time and require high temperatures, and the reagents must be prepared often [20, 25, 30]. Hence, the KOCH_3_/HCl method under milder conditions may not be sufficient to obtain complete methylation, and these factors may explain the poor results observed for UFAs and TFAs in comparison with other methods. However, this method is faster, easy to use, less expensive, and more environmentally friendly than the TMS-DM method. Thus, the KOCH_3_/HCl method could be more applicable for routine analysis and study of the general composition of FAs in some food samples.

In contrast, the TMS-DM method showed the best balance between recovery and variation values, especially for the *cis*/*trans* UFAs, when compared to the second method. It also had the lowest intraday and interday variation for most FAs and TFAs. This finding is most likely because of the use of TMS-DAM as an alternative to an acid catalyst. TMS-DM is an ideal derivatization reagent and a convenient alternative source of diazomethane, which is known to be safer to handle and more stable [40, 44]. It converts carboxylic acids to methyl esters in high yields with short incubation times and forms few by-products (N_2_) [39]. Furthermore, the esterification by TMS-DAM has been reported to be effective and accurate for the analysis of FA isomers in various food samples, such as the analysis of *cis*/*trans* PUFAs in seafood [31] and CLA isomers in ruminant meat tissues [27, 32]. Otherwise, the base catalyst (NaOCH_3_) is a useful reagent for the fast transmethylation of FAs linked to TAG, is stable for a long time, and performs better on lipids rich in FAs with PUFAs, TFAs, and CLA [14, 18, 19, 35]. In addition to all these advantages, TMS-DM and NaOCH_3_ do not change the original FA distribution or alter the geometric configuration of the double bonds during the transformation reactions [18, 19, 27, 31]. Artifacts are not produced when NaOCH_3_ is used as a transesterification agent [30, 35] and TMS-DM does not produce the methoxy artifacts associated with base-catalyzed method [31, 32, 39, 40]. Accordingly, this method resulted in less FA and TFA losses than using the acid-catalyzed methylation method as well as a complete methylation of FFAs.

In general, both methods were found to be suitable for the determination of FA profiles and for quantifying relatively different levels of *cis *and *trans* FAs in several bakery products, such as biscuits, cookies, crackers, wafers, cakes, and bread. However, no single method can fulfill the derivatization requirement for all types of food samples. Consequently, a good alternative could be to have both methods available for use in the laboratory. The KOCH_3_/HCl method is ideal for the routine and fast analysis of samples that do not contain a complex mixture of FAs and TFAs, and the TMS-DM method is ideal for a more thorough analysis of rich *cis*/*trans* UFA samples, such as bakery, dairy, and ruminant meat products, and for monitoring low levels of FAs and TFAs as well as controlling labeling authenticity.

For both methods, the appropriate use of an IS during the procedure might partially correct the recovery values for both methods and compensate for any partial hydrolysis that may occur during the course of the reactions [27]. Moreover, according to Eder [45] and Christie and Han [15], the extraction of FAMEs should be performed more than one time for complete recovery, which may assist in improving the efficiency and accuracy of the performance by increasing the recovery values for both methods. Otherwise, lipid oxidation involves the attack of free radicals (formation by oxygen) to adjacent positions of double bonds [27], and these factors are controlled in the TMS-DM method with the addition of the antioxidant agent BHT during FAME extraction and before storage, whereas the KOCH_3_/HCl method has been originally validated without using antioxidants and there was no indication for the need to use antioxidants with this method.

## 4. Conclusions

The results of the absolute concentration of most FAs in several bakery products, such as biscuits, cookies, crackers, wafers, cakes, and bread, by GC-FID using two derivatization procedures (KOCH_3_/HCl and TMS-DM), have good agreement. However, higher concentrations of all *cis* and *trans* UFAs were detected when following the TMS-DM method compared to the KOCH_3_/HCl method, and the results of the relative proportion of the TFAs were significantly higher when estimated using the TMS-DM method compared to the KOCH_3_/HCl method. The results from the KOCH_3_/HCl method showed the lowest %*R* values, especially for *cis*/*trans* UFAs, and it almost had acceptable intraday and interday variation values, except for the TFAs. Overall, this method, under the milder conditions used, may not be sufficient to obtain complete methylation. Nevertheless, this method is faster, less expensive, and more environmentally friendly than the TMS-DM method. In contrast, the TMS-DM method had better recovery values and the lowest intraday and interday variation values compared to the other method. It presents the best balance combination between recovery and variation values, especially for UFAs and TFAs. It is likely that using NaOCH_3_ along with TMS-DM as an alternative to the acid catalyst in this method could result in a decrease in the loss of FAs and TFAs as well as a complete methylation of FFAs when compared to the acid-catalyzed methylation method. Accordingly, the KOCH_3_/HCl method could be appropriate for the routine analysis and compositional study of FAs in some food samples, whereas the TMS-DM method could be available for more accurate quantification of food samples containing a complex mixture of FAs and TFAs.

## Figures and Tables

**Table 1:** The mean of the absolute (g FA 100 g^−1^ sample) and relative (% of total identified FA) content of each FA determined using the KOCH_3_/HCl method.

Fatty acids	Detected concentration [g FA 100 g^−1^], (% Total FA)							
	Samples							
	A	B	C	D	E	F	G	H
C12:0	—	—	[10.35], (42.23)	[1.48], (6.48)	[10.09], (39.80)	—	[5.04], (31.11)	—
C14:0	[0.16^a^], (1.23^a^)	[0.27^a^], (1.88)	[2.38^a^], (11.06)	[0.65], (2.89)	[3.94^a^], (15.55)	—	[0.75^a^], (4.63)	—
C16:0	[9.21], (48.35)	[9.28], (43.43)	[2.53], (10.70)	[8.87], (38.05)	[4.16], (16.41^a^)	[9.76], (48.70^a^)	[5.91^a^], (36.48^a^)	[7.78], (41.18)
C18:0	[0.79^a^], (4.95)	[0.84^a^], (4.96)	[3.65^a^], (17.24)	[1.65^a^], (8.59)	[1.07^a^], (4.22)	[0.94], (4.69)	[0.31^a^], (1.91^a^)	[0.87^a^], (4.61)
C18:1 *tran*-9	—	—	[1.21^a^], (5.11^a^)	—	[0.32^a^], (1.01^a^)	[0.12^a^], (0.59^a^)	[0.45^a^], (2.77^a^)	[1.08^a^], (4.90^a^)
C18:1	[6.62^a^], (38.70^a^)	[8.97^a^], (41.04^a^)	[1.10], (5.65)	[7.94^a^], (34.83^a^)	[2.02^a^], (7.97^a^)	[6.73^a^], (34.21^a^)	[1.83^a^], (11.30^a^)	[7.86^a^], (41.06^a^)
C18:2 *trans*-9,12	—	[0.02^a^], (0.95^a^)	—	[0.03^a^], (0.11^a^)	[0.14^a^], (0.48^a^)	[0.09^a^], (0.43^a^)	—	[0.12^a^], (0.60^a^)
C18:2	[1.17^a^], (6.97^a^)	[1.41^a^], (7.29^a^)	[0.10^a^], (0.42^a^)	[1.72^a^], (8.76^a^)	[0.82^a^], (2.84^a^)	[2.18], (10.90^a^)	[0.81^a^], (5.0^a^)	[1.34^a^], (7.09^a^)
C18:3	—	[0.06^a^], (0.27^a^)	[0.04^a^], (0.17^a^)	[0.07^a^], (0.28^a^)	[0.45^a^], (1.55^a^)	—	—	—

^a^Significant differences (*P* ≤ 0.05); [—] not detected.

**Table 2:** The mean of the absolute (g FA 100 g^−1^ sample) and relative (% of total identified FA) content of each FA determined using the TMA-DM method.

Fatty acids	Detected concentration [g FA 100 g^−1^], (% Total FA)							
	Samples							
	A	B	C	D	E	F	G	H
C12:0	—	—	[9.77], (39.65)	[1.49], (6.17)	[10.95], (37.85)	—	[4.82], (29.75)	—
C14:0	[0.20^b^], (1.09^b^)	[0.36^b^], (1.62)	[2.89^b^], (12.23)	[0.69], (2.86)	[4.73^b^], (16.36)	—	[0.81^b^], (5.04)	—
C16:0	[8.95], (47.08)	[9.34], (41.70)	[2.29], (9.69)	[8.76], (36.08)	[4.20], (14.54^b^)	[9.41], (45.04^b^)	[4.97^b^], (30.70^b^)	[8.61], (39.14)
C18:0	[0.86^b^], (4.50)	[0.99^b^], (4.43)	[4.18^b^], (17.70)	[2.06^b^], (8.44)	[1.23^b^], (4.25)	[1.05], (5.04)	[0.37^b^], (2.30^b^)	[1.12^b^], (5.09)
C18:1 *tran*-9	—	—	[1.83^b^], (7.75^b^)	—	[0.45^b^], (1.54^b^)	[0.19^b^], (0.89^b^)	[0.56^b^], (3.47^b^)	[1.74^b^], (7.90^b^)
C18:1	[7.22^b^], (37.91^b^)	[9.56^b^], (42.70^b^)	[1.13], (4.79)	[8.87^b^], (36.47^b^)	[2.85^b^], (9.86^b^)	[7.97^b^], (38.14^b^)	[2.19^b^], (13.51^b^)	[8.63^b^], (39.22^b^)
C18:2 *trans*-9,12	—	[0.04^b^], (0.19^b^)	—	[0.05^b^], (0.17^b^)	[0.33^b^], (1.13^b^)	[0.14^b^], (0.66^b^)	—	[0.18^b^], (0.85^b^)
C18:2	[1.83^b^], (9.62^b^)	[2.01^b^], (8.99^b^)	[0.13^b^], (0.57^b^)	[2.29^b^], (9.43^b^)	[1.24^b^], (4.28^b^)	[2.14], (10.23^b^)	[0.99^b^], (6.17^b^)	[1.75^b^], (7.95^b^)
C18:3	—	[0.08^b^], (0.35^b^)	[0.05^b^], (0.22^b^)	[0.09^b^], (0.38^b^)	[0.56^b^], (2.06^b^)	—	—	—

^b^Significant differences (*P* ≤ 0.05); [—] not detected.

**Table 3:** Correlation coefficients between the KOCH_3_/HCl method and TMS-DM method.

Fatty acids	Correlation coefficients (*r*) for g/100 g
C12:0	0.91
C14:0	0.89
C16:0	0.99
C18:0	0.95
C18:1 *trans*-9	0.96
C18:1	0.98
C18:2 *trans*-9,12	0.86
C18:2	0.94
C18:3	0.89

**Table 4:** The recovery percentage (*R*%, calculated from four samples studied) at two addition levels for both methods employed.

Sample	Std	*R*% for KOCH_3_/HCl, (*R*% for TMS-DM)								
		Fatty acids								
		C12:0	C14:0	C16:0	C18:0	C18:1 t9	C18:1	C18:2 t9, t12	C18:2	C18:3
A	1	106.8	87.7	110.8	97.3	95.9	97.8	86.9	93.2	99.5
		(104.3)	(92.8)	(104.9)	(97.9)	(102.0)	103.12	(98.9)	(95.8)	(98.8)
	2	105.9	87.2	109.4	95.5	92.2	94.0	83.7	90.8	98.1
		(103.2)	(89.6)	(105.8)	(94.3)	(98.7)	(104.9)	(93.8)	(92.3)	(96.0)

B	1	98.1	96.8	112.4	91.5	93.4	97.1	91.0	88.7	104.1
		(96.7)	(101.7)	(106.0)	(89.8)	(95.2)	(103.3)	(97.0)	(94.6)	(105.6)
	2	96.5	95.8	106.3	92.4	91.4	94.1	88.7	83.4	101.5
		(95.4)	(98.3)	(105.4)	(90.7)	(92.1)	(101.8)	(95.1)	(93.4)	(103.1)

C	1	92.4	93.61	106.9	93.5	83.7	97.75	83.6	85.9	103. 6
		(93.4)	(100.7)	(105.2)	(89.8)	(92.3)	(102.2)	(93.7)	(92.6)	(104.5)
	2	91.1	91.8	104.1	91.5	83.9	97.1	82.6	84.2	104.0
		(91.2)	(99.2)	(103.2)	(89.2)	(91.2)	(104.2)	(89.5)	(91.2)	(106.2)

D	1	104.1	97.7	102.1	96.5	90.9	94.0	86.6	101.2	89.0
		(101.9)	(102.6)	(100.7)	(98.0)	(98.8)	(99.1)	(103.4)	(104.1)	(97.3)
	2	98.1	96.8	96.1	96.5	87.9	93.1	84.0	98.2	85.0
		(98.4)	(101.2)	(96.5)	(97.2)	(94.3)	(98.2)	(98.4)	(104.2)	(95.2)

^a^ *R*: recovery; Std: standard solution; t: *trans* fatty acids.

**Table 5:** Intraday variation (RSD, %) for four studied samples by both methods employed.

Fatty acids	Sample (*n* = 4, RSD %)^a^							
	A		B		C		D	
	i	ii	i	ii	i	ii	i	ii
C12:0	2.48	2.04	1.98	1.75	2.95	1.49	2.55	2.48
C14:0	3.21	3.62	2.60	1.50	1.77	1.85	3.13	1.79
C16:0	2.14	1.19	2.05	0.32	2.90	2.28	4.32	0.98
C18:0	2.58	0.92	1.88	0.59	3.07	3.88	2.34	2.03
C18:1 *trans*-9	5.03	1.14	4.23	2.02	6.27	2.17	5.92	3.01
C18:1	3.44	2.26	1.10	0.89	3.55	1.99	1.90	1.27
C18:2 *trans*-9,12	6.84	2.56	5.41	1.01	4.68	2.01	6.77	2.99
C18:2	4.06	1.56	3.77	1.89	2.60	2.55	3.15	0.93
C18:3	2.58	3.02	4.42	2.40	0.99	0.86	4.67	2.11

^a^RSD: relative standard deviation; (i) the KOCH_3_/HCl method; (ii) the TM-SD method.

**Table 6:** Interday variation (RSD, %) for four studied samples by both methods employed.

Fatty acids	Sample (*n* = 3, RSD %)^a^							
	A		B		C		D	
	i	ii	i	ii	i	ii	i	ii
C12:0	3.44	2.98	4.12	2.05	3.50	3.44	3.92	3.35
C14:0	4.21	5.60	3.60	5.15	4.29	4.12	4.51	5.20
C16:0	3.14	2.11	2.05	1.03	3.80	2.98	3.19	2.55
C18:0	2.58	4.72	3.88	2.99	2.58	1.44	2.98	4.01
C18:1 *trans*-9	6.03	3.20	5.23	2.91	5.44	3.23	6.29	2.88
C18:1	3.44	3.13	3.10	1.87	4.91	4.33	2.56	3.51
C18:2 *trans*-9,12	7.04	4.14	6.41	3.21	7.11	2.92	6.74	3.75
C18:2	2.06	1.81	4.77	3.80	4.67	3.35	5.14	2.70
C18:3	3.58	5.42	4.42	4.73	5.01	5.11	3.65	4.99

^a^RSD: relative standard deviation; (i) the KOCH_3_/HCl method; (ii) the TM-SD method.
